# Visual Outcomes, Intraocular Pressure Changes, and Complications Following Nd:YAG (Neodymium-Doped Yttrium Aluminum Garnet) Laser Posterior Capsulotomy for Posterior Capsular Opacification: A Prospective Clinical Study

**DOI:** 10.7759/cureus.114720

**Published:** 2026-08-18

**Authors:** Sujata Charel, Nikhil Gore

**Affiliations:** 1 Ophthalmology, Sumandeep Vidyapeeth Deemed to be University, Vadodara, IND

**Keywords:** capsulotomy, cystoid macular edema, intraocular pressure, iritis, nd:yag laser, retinal detachment

## Abstract

Aims and backgrounds

Posterior capsular opacification (PCO) is the most common long-term complication after cataract surgery. It leads to decreased contrast sensitivity, distracting glare, and impaired vision. The neodymium-doped yttrium aluminum garnet (Nd:YAG) laser posterior capsulotomy is the de facto standard for non-invasive treatment of visually significant PCO. It enables rapid vision recovery and is considered a gold standard procedure. Nevertheless, complications including increased intraocular pressure (IOP), cystoid macular edema (CME), and retinal detachment are possible following the procedure.

Methods

Prospective observational research was done at a tertiary care center in Vadodara, Gujarat. Patients with PCO undergoing Nd:YAG laser capsulotomy were included based on defined inclusion and exclusion criteria. Intraocular pressure (IOP), slit lamp examination, dilated fundus examination, uncorrected and best-corrected visual acuity (UCVA, BCVA), and intraocular pressure (IOP) were all part of the pre- and post-procedure measurements. Complications such as CME, retinal detachment, iritis, and IOP rise were recorded. The relationship between total laser energy used and IOP changes was also assessed.

Results

Most patients achieved rapid, significant visual improvement. Transient IOP elevations were infrequent and medically manageable, with higher risks linked to increased cumulative laser energy. Severe complications like CME were rare.

Conclusion

Nd:YAG laser capsulotomy is a safe, effective outpatient procedure. Optimal energy settings and post-operative monitoring are vital to maximize recovery and minimize complications.

Clinical significance

To ensure optimal outcomes and minimize risks, clinicians should use the minimum effective laser energy, ensure precise focusing, and routinely monitor post-procedural intraocular pressure.

## Introduction

Despite advances in modern cataract surgery, cataract remains the leading global cause of reversible blindness. While phacoemulsification with intraocular lens (IOL) implantation has vastly improved patient outcomes, posterior capsule opacification (PCO) remains the most prevalent long-term complication [1]. Driven by the proliferation, migration, and differentiation of surviving lens epithelial cells, PCO leads to visual decline, glare, and diminished contrast sensitivity [2].

The non-invasive neodymium-doped yttrium aluminum garnet (Nd:YAG) laser posterior capsulotomy is the gold standard for treating visually significant PCO. The laser delivers precisely focused, short-duration high-energy pulses that create a central opening in the opacified posterior capsule through photodisruption, restoring an unobstructed optical pathway to the retina and improving visual function. By precisely clearing the opaque capsule, it restores the optical axis and improves visual acuity [3]. Because it avoids intraocular manipulation, it has largely replaced surgical capsulotomy and significantly reduced the severe intraocular risks associated with surgical intervention [4].

Although generally safe, Nd:YAG capsulotomy carries potential complications that require careful management [5]. Visual recovery is typically prompt, but outcomes can be hindered by procedural complications, PCO morphology, or pre-existing ocular comorbidities [6]. The most common early complication is an acute rise in intraocular pressure (IOP), resulting from trabecular meshwork obstruction by liberated capsular debris or inflammatory cells [7]. While usually transient, significant spikes pose a risk to susceptible patients. Other early complications such as anterior chamber inflammation may occur. Late complications include cystoid macular edema (CME), caused by laser-induced inflammation disrupting the blood-retinal barrier [8], anterior uveitis, intraocular lens pitting, and, rarely, retinal detachment linked to vitreous destabilization [9]. Crucially, the total laser energy utilized directly influences these risks; higher cumulative energy increases debris release and intraocular inflammation, thereby elevating complication rates [10].

Given the high volume of Nd:YAG capsulotomies performed globally, continuous evaluation of its efficacy and safety profile is essential. Therefore, this study aimed to evaluate the visual outcomes and complications following Nd:YAG laser capsulotomy for PCO. By systematically assessing visual acuity (VA), intraocular pressure (IOP) changes, procedure-related complications, and the direct correlation between total laser energy and IOP spikes, this research provides vital insights to optimize patient selection, refine laser parameters, and minimize postoperative risks. The present study primarily aimed to evaluate the effect of Nd:YAG laser posterior capsulotomy on visual acuity and intraocular pressure in patients with posterior capsule opacification following uncomplicated cataract surgery. Other secondary objectives were to assess changes in IOP at 1 hour after the procedure, at 1 week, and at 14 days; to determine the incidence of procedure-related complications during follow-up; and to evaluate the association between total laser energy used and post-procedural IOP elevation.

## Materials and methods

Ethical consideration

The Sumandeep Vidyapeeth Institutional Ethics Committee approved the study (approval number: SVIEC/ON/MEDI/BNPG23/AUGUST/24/2, dated: August 3, 2024). Participants provided written informed consent for voluntary involvement. Strict patient confidentiality was maintained, and standard ethical guidelines were strictly followed throughout the research. The study was carried out from the date of approval by the Institutional Ethics Committee until the desired sample size was attained (August 2024-January 2025).

Study design and setting

A prospective observational study at a tertiary care hospital in Vadodara evaluated 120 patients. The sample size was calculated using the standard sample size formula, $N = \frac{Z^{2} \, p (1-p)}{e^{2}}$, where N = required sample size; Z = standard normal variate at a 95% confidence interval = 1.96; P = anticipated proportion = 50% = 0.50; and e = allowable error/absolute precision = 9% = 0.09.

Substituting the values:

n = (1.96² × 0.50 × (1 - 0.50)) / (0.09²)

n = (3.8416 × 0.50 × 0.50) / 0.0081

n = 0.9604 / 0.0081

n = 118.56

n ≈ 119

Considering feasibility and rounding, the final sample size was taken as 120 patients are undergoing Nd:YAG laser capsulotomy for PCO. The cohort featured a balanced gender and eye laterality distribution.

Participants

A total of 120 patients who had undergone cataract surgery with visually significant PCO were included. Patients with significant corneal/retinal pathology, recurrent iridocyclitis, or uncontrolled glaucoma were excluded.

Procedure

Following comprehensive baseline ocular evaluations, patients underwent cruciate capsulotomy using a Q-switched Nd:YAG laser (1064 nm) starting at 2.5 mJ [2]. Post-procedure regimens included topical timolol 0.5% two times a day and a topical antibiotic-steroid combination of moxifloxacin (0.5%) and prednisolone (1%) three times a day to mitigate IOP spikes and inflammation [3].

Data collection

Follow-ups occurred at 1 hour, 1 week, and 2 weeks to assess visual acuity, IOP by applanation tonometry, and complications (e.g., CME by optical coherence tomography). Total laser energy and shot count were recorded.

Statistical analysis

Data were analyzed utilizing standard statistical software (t-tests, ANOVA, Chi-square), with p < 0.05 considered statistically significant.

## Results

Study demographics

The study evaluated 120 participants undergoing Nd:YAG laser capsulotomy for posterior capsular opacification (PCO). The mean age was 61.50 ± 10.02 years (range: 45-75), representing a predominantly middle-aged and elderly population. The gender distribution was balanced, with 50.8% (n=61) males and 49.2% (n=59) females. Eye involvement was nearly equal, affecting the right eye in 51.7% (n=62) of cases and the left eye in 48.3% (n=58).

Pre-procedure clinical profile

Baseline vision was reduced in all patients, with the majority at 20/120 (37.5%), followed by 20/200 (35.0%) and 20/80 (27.5%). Slit lamp examination (SLE) identified Elschnig pearls as the most common presentation (40.0%), followed by the fibrous type (31.7%) and Soemmering ring (28.3%). Mean baseline IOP was normal at 15.98 ± 1.21 mmHg (range: 14-18 mmHg). The majority of patients were myopic (70.0%), with 29.2% hypermetropic and 0.8% emmetropic. Energy per shot was mean of 1.75 ± 0.15 mJ.

Laser parameters

The laser settings utilized were within standard and clinically acceptable limits. The mean number of shots was 14.71 ± 7.39 (range: 5-35). The mean total energy was 25.48 ± 12.45 mJ (range: 10-60 mJ), with most treated in the 10-30 mJ r (Table 1).

**Table 1:** Laser parameters used during Nd:YAG laser capsulotomy (n = 120) Nd:YAG: neodymium-doped yttrium aluminum garnet.

Parameter	N	Mean	SD	Minimum	Maximum
Number of shots	120	14.71	7.39	5	35
Energy per shot (mJ)	120	1.75	0.15	1.50	2.00
Total energy (mJ)	120	25.48	12.45	10	60

Post-procedure outcomes

**Figure 1:**
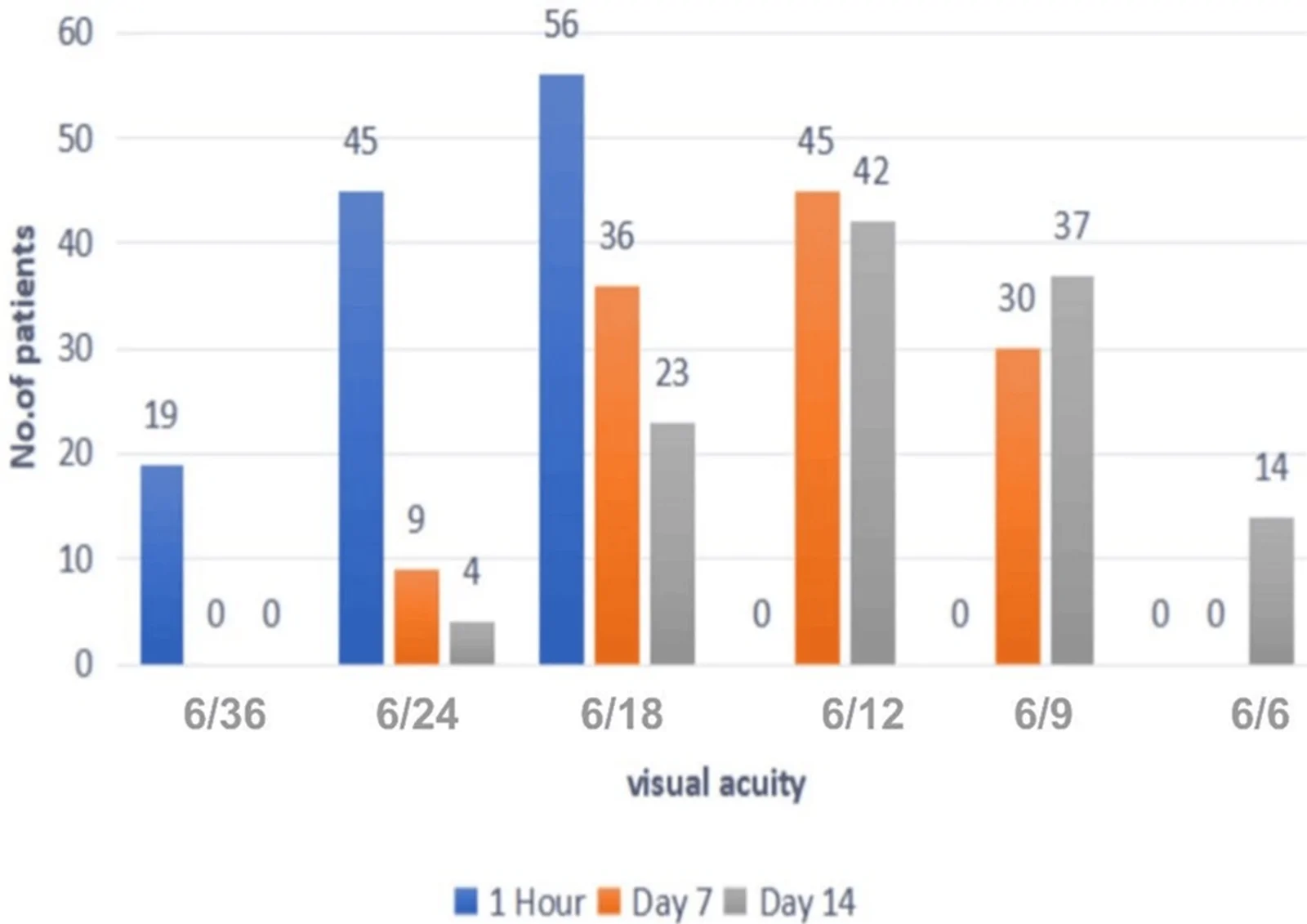
Post-procedure best-corrected visual acuity (BCVA) at different follow-up intervals (n = 120)

Patients experienced progressive and sustained improvement in visual acuity across all follow-up intervals (Figure 1).

The majority of the patient recovered visual acuity within 1 hour was 6/18 (46.7%). On Day 7, vision improved further, with the majority at 6/12 (37.5%) and 6/9 (25.0%), Day 14 was marked improvement continued, with 35.0% at 6/12, 30.8% at 6/9, and 11.7% achieving 6/6 vision. Poor vision (6/36 and 6/24) steadily decreased.

Anatomical clarity

A clear visual axis was achieved in 55.0% of patients at 1 hour, peaking at 62.5% by Day 7, and stabilizing at 59.2% by Day 14. Mild inflammatory cells and residual capsular tags were observed initially but remained stable or decreased over the follow-up period (Table 2).

**Table 2:** Post-procedure slit lamp examination (SLE) findings at different follow-up intervals (n = 120)

SLE findings	1 hour n (%)	Day 7 n (%)	Day 14 n (%)
Clear visual axis	66 (55.0)	75 (62.5)	71 (59.2)
Mild cells	19 (15.8)	26 (21.7)	25 (20.8)
Residual tags	35 (29.2)	19 (15.8)	24 (20.0)
Total	120 (100)	120 (100)	120 (100)

Refractive stability

**Figure 2:**
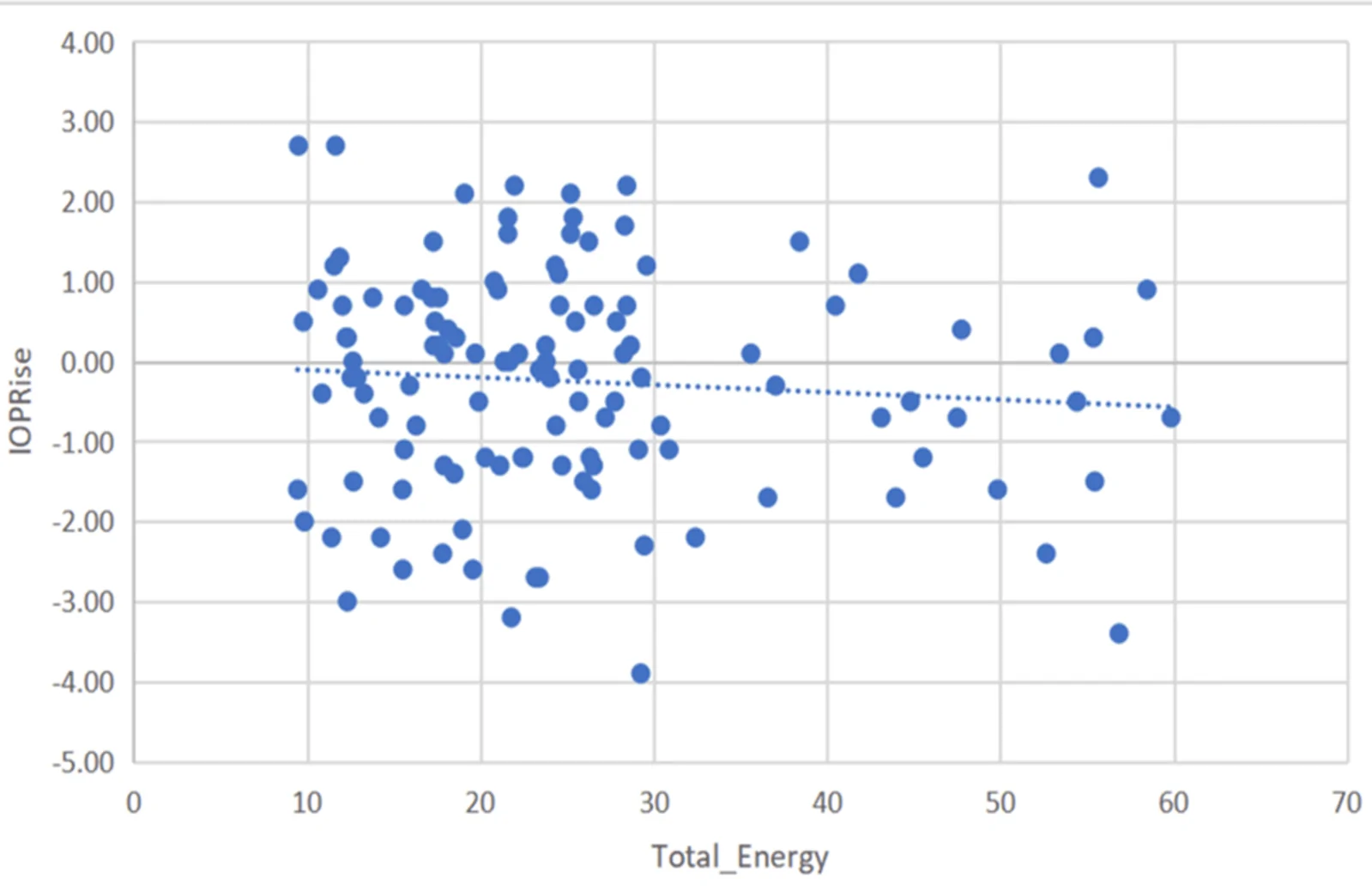
Correlation between total energy and rise in intraocular pressure (IOP) (n = 120)

Refractive status remained relatively stable post-procedure, with myopia persisting as the dominant refractive error (65.0% to 68.3% across follow-ups) and no significant alterations induced by the laser. The paired comparison of intraocular pressure (IOP) at different time intervals (n = 120) was assessed (Table 3), and correlation between total energy and rise in intraocular pressure (IOP) was evaluated (Figure 2).

**Table 3:** Paired comparison of intraocular pressure (IOP) at different time intervals (n = 120)

Comparison	Mean difference	t value	p value
Pre vs 1 hour	-2.58	-30.35	<0.001
1 hour vs Day 7	2.59	14.19	<0.001
Day 7 vs Day 14	0.32	2.50	0.014

There was a statistically significant transient spike in IOP at 1 hour post-procedure (p < 0.001), followed by a significant decrease in IOP toward baseline by Day 7 (p < 0.001), and IOP stabilized by Day 14. So, there was no clinically meaningful or statistically significant correlation between the total laser energy used and the initial rise in IOP (r = -0.08, p = 0.372). The procedure demonstrated a highly favorable safety profile. Complications occurred in 45.8% of cases but were predominantly mild and self-limiting. The complications included transient IOP rise: 13.3% (n = 16), iritis: 10.0% (n = 12), pain: 7.5% (n = 9), IOL pitting: 5.8% (n = 7), nausea: 5.0% (n = 6), cystoid macular edema (CME): 4.2% (n = 5), and retinal detachment: 0.0% (n = 0).

## Discussion

In the present study, we achieved a favorable safety profile using a lowest mean total energy of 25.48 mJ (averaging 1.75 mJ per shot). The mean number of shots was 14.71 ± 7.39, ranging from 5 to 35 shots. The energy per shot aligns closely with Kaur et al. (1.5-2.5 mJ) [11], but is narrower and lower than the range reported by Matin et al. (1-4.5 mJ) [12]. The total energy used was significantly lower than the totals reported by Kolli H et al. (average 79 mJ) [13] and Joshi and Doble (medians of 42.62 mJ and 46.95 mJ) [14].

Patients presented with a normal and stable mean baseline IOP of 15.98 ± 1.21 mmHg. Establishing a normal baseline is a standard practice for evaluating post-procedure complications. The study's baseline mean closely matches the initial 15.8 ± 2.3 mmHg level reported by Kaur et al. [11].

There was a statistically significant, yet transient, rise in IOP 1 hour post-procedure. Pressures significantly decreased and stabilized by Day 7, with minor reductions continuing to Day 14. In total, 13.3% of patients experienced this transient spike. The transient nature of the IOP spike is well-documented, with the 13.3% incidence rate aligning closely with the 12% rate observed by Kaur et al. [11]. Similar temporary elevations requiring minimal or short-term management before returning to near-baseline levels within a week were also reported by Vaidhya et al. [15] and Joshi and Doble [14].

The study found no significant correlation between the total laser energy used (mean: 25.48 ± 12.45 mJ) and subsequent intraocular pressure (IOP) rise (r = -0.08, p = 0.372). This indicates that within this energy range, IOP spikes are not strictly energy-dependent and are likely multifactorial (e.g., influenced by capsular debris, inflammation, and individual ocular susceptibility). This lack of direct correlation is supported by Kolli et al. [13], who observed no complications despite using significantly higher energies (average 79 mJ). In contrast, other studies highlight varying ocular responses: Joshi and Doble [14] and Kaur et al. [11] reported rare or transient IOP spikes at different energy thresholds, while Mbaga et al. [16] identified elevated energy as a risk factor for suboptimal visual outcomes.

Elschnig pearls were the predominant posterior capsular opacification (PCO) morphology (40.0%), followed by fibrous PCO (31.7%) and Soemmering ring (28.3%). The prevalence of Elschnig pearls is consistent with previous reports by Dhakne et al. [17] and Vaidhya et al. [15]. Severe PCO at this baseline is a known risk factor for suboptimal post-capsulotomy visual outcomes.

All 120 patients experienced moderate to severe visual impairment prior to intervention, with best-corrected visual acuity (BCVA) levels at 6/36 (37.5%), 6/60 (35.0%), and 6/24 (27.5%). No patient presented with a BCVA of 6/18 or better. These significant visual impairments, which remains the primary indication for Nd:YAG laser capsulotomy, closely correspond with the 6/36 average reported by Kaur et al. [11] and align with the significant baseline vision reductions observed by Dhakne et al. [17] and Mbaga et al. [16].

The study demonstrated progressive and marked visual rehabilitation following Nd:YAG laser capsulotomy. By Day 14, 77.5% of patients achieved a best-corrected visual acuity (BCVA) of 6/12 or better (with 11.7% reaching 6/6). These significant visual gains are highly consistent with existing literature. Similar progressive improvements in visual acuity were reported by Dhakne et al. [17], a strong return to 6/9 vision was noted by Kaur et al. [11], and a high overall improvement rate (82.7% of patients) was observed by Mbaga et al. [16].

Myopia was the predominant refractive error both before (70.0%) and after (68.3% at Day 14) the Nd:YAG laser capsulotomy. The procedure did not cause any major refractive shifts, indicating that patients still require refractive correction (such as glasses) following a successful capsulotomy. The stability of the refractive status confirms that post-procedural visual improvements are due to the optical clearing of the posterior capsule rather than a change in refraction. This aligns with findings by Lu et al. [18], who reported significant improvements in visual quality parameters following the procedure without major refractive alterations.

The procedure produced a satisfactory anatomical outcome, with the majority of patients achieving a clear central visual axis (peaking at 62.5% on Day 7 and remaining stable at 59.2% by Day 14). Mild inflammation (cells noted in 20.8% of cases) and residual capsular tags (20.0%) persisted through Day 14. Clinical iritis was also recorded in 10.0% of patients. The persistent residual tags likely account for the incomplete visual recovery seen in a small proportion of patients. Matin et al. [12] highlighted the importance of standard slit-lamp examinations to rule out underlying ocular diseases. In contrast to the mild inflammation and iritis observed here, Kolli et al. [13] reported an absence of post-laser issues or worsening inflammation in their specific uveitis series.

Complications were generally mild and manageable, occurring in 45.8% of patients. The most frequently encountered complications were transient intraocular pressure (IOP) rise (13.3%), iritis (10.0%), pain (7.5%), intraocular lens (IOL) pitting (5.8%), nausea (5.0%), and cystoid macular edema (4.2%). Notably, no cases of retinal detachment occurred. Although the overall complication rate was higher than the 13.76% reported by Matin et al. [12] and the complete absence of complications noted by Lighthizer et al. [19], the specific types of mild complications and the lack of severe adverse events align well with existing research. The 13.3% transient IOP spike closely matches the 12% rate found by Kaur et al. [11], and similar minor issues like IOL pitting and elevated IOP were also documented by Dhakne et al. [17] and Joshi and Doble [14].

Nd:YAG laser capsulotomy proved to be a highly effective and safe outpatient intervention with manageable complications. It yielded rapid visual recovery, with 77.5% of patients achieving a best-corrected visual acuity (BCVA) of 6/12 or better by Day 14, a stark contrast to the universal baseline impairment (ranging from 6/24 to 6/60). This high rate of visual rehabilitation strongly aligns with established research. Similar marked improvements following the procedure have been widely documented, including a 99% improvement rate reported by Lighthizer et al. [19] and an 82.7% improvement rate noted by Mbaga et al. [16]. Substantial visual gains to 6/12 or 6/9 were also echoed in findings by Dhakne et al. [17] and Kaur et al. [11].

Strengths of the study

This study evaluated a well-sized cohort of 120 patients with balanced demographics and specific serial follow-ups at 1 hour, Day 7, and Day 14. It also included well-documented laser parameters (shots, energy per shot, and total energy) and statistically analyzed their correlation with changes in intraocular pressure (IOP). It also confirms that Nd:YAG laser capsulotomy is a highly effective and rapid outpatient procedure for restoring functional vision in posterior capsular opacification (PCO) and demonstrates a favorable safety profile when appropriate precautions and post-procedure monitoring are undertaken. The significant early IOP spike (at 1 hour) and potential for minor complications mandate routine post-procedural monitoring. The strengths of the present study include its prospective study design, which enabled standardized pre- and post-procedural assessment of patients. The study comprehensively evaluated multiple clinically relevant outcomes, including visual acuity, intraocular pressure, procedure-related complications, and the association between total laser energy and post-procedural IOP elevation. Uniform assessment methods and predefined follow-up visits further enhanced the reliability of the findings.

Clinical recommendations

Nd:YAG laser capsulotomy is recommended as the primary treatment for visually significant PCO following a detailed pre-procedure ophthalmic evaluation. It utilizes the lowest effective laser energy. Early IOP checks are mandatory, short-term anti-inflammatories should be considered, and patients should be educated on warning signs of severe complications (e.g., flashes, floaters, and vision loss).

Study limitations

The short 14-day follow-up misses late-onset complications (e.g., late cystoid macular edema, retinal tears). The present study lacked a control group, did not stratify outcomes by PCO severity/morphology, was limited to a single center, and relied exclusively on Snellen visual acuity rather than broader visual quality indices such as contrast sensitivity or glare testing.

Future research directions

Extended follow-ups (months to a year) are needed to assess delayed complications and long-term safety. Future studies should incorporate objective visual quality assessments (wavefront analysis, contrast sensitivity) and patient-reported outcomes. More research is required for high-risk groups (e.g., glaucoma, high myopia, uveitis) and to evaluate the efficacy of prophylactic medications. The procedure provided rapid and sustained visual recovery without causing major refractive shifts. By Day 14, 77.5% of the 120 patients achieved a best-corrected visual acuity (BCVA) of 6/12 or better.

## Conclusions

The findings reinforce Nd:YAG laser capsulotomy as a highly effective, safe outpatient intervention for PCO. To optimize clinical outcomes, it is recommended to use the lowest effective laser energy, prescribe short-term topical anti-inflammatories, and mandate routine post-procedural IOP monitoring, particularly in the immediate 1-hour window, to manage transient pressure elevations effectively. To ensure optimal outcomes and minimize risks, clinicians should use the minimum effective laser energy, ensure precise focusing, and routinely monitor post-procedural intraocular pressure.
